# Gastric Cancer: Global Epidemiology, Risk Factors, *Helicobacter pylori* Eradication, and Risk-Based Screening

**DOI:** 10.3390/jcm15218530

**Published:** 2026-11-02

**Authors:** Sara Jouya, Mai Abdel Haleem A. Abusalah, Zahra Shahabinia, Afrooz Mazidimoradi, Leila Allahqoli, Hamid Salehiniya, Do-Youn Lee

**Affiliations:** 1 Health Deputy, Birjand University of Medical Sciences, Birjand 9717853577, Iran; sarahjouya@gmail.com; 2 Department of Medical Laboratory Sciences, Faculty of Allied Medical Sciences, Al-Ahliyya Amman University, Amman 19328, Jordan; 3 Geriatric Health Research Center, Birjand University of Medical Sciences, Birjand 9717853577, Iran; 4 Health Assistant Department, Shiraz University of Medical Sciences, Shiraz 7193711351, Iran; 5 Faculty of Health Sciences, Cyprus International University, Nicosia 99258, Cyprus; 6 Department of Epidemiology and Biostatistics, School of Health, Social Determinants of Health Research Center, Birjand University of Medical Sciences, Birjand 9717853577, Iran; 7 College of General Education, Kookmin University, Seoul 02707, Republic of Korea

**Keywords:** gastric cancer, epidemiology, risk factors, *Helicobacter pylori*, eradication, endoscopic screening, prevention

## Abstract

**Background:** Declining age standardized rates conceal differences in gastric cancer by age, sex, anatomical subsite, histological type, and region. This review examines these patterns alongside risk factors, *Helicobacter pylori* eradication, screening, and surveillance. **Methods:** PubMed, Web of Science, and Scopus were searched iteratively for literature available through January 2026. Google Scholar and reference lists were used to identify additional relevant sources. One reviewer selected sources, and findings were interpreted qualitatively according to study design, population, confounding, precision, and geographic applicability. **Results:** Standardized rates have fallen in many countries, while absolute case numbers continue to rise. Increases reported in some younger groups do not establish a uniform global trend because age definitions and registry coverage differ. *Helicobacter pylori* is a major causal factor, but mucosal damage, hereditary susceptibility, and other exposures affect risk. Randomized trials support eradication for reducing primary and metachronous cancer incidence. Screening associations with earlier diagnosis and lower mortality are largely observational and concentrated in East Asia. **Conclusions:** Prevention requires treatment of *Helicobacter pylori* and confirmation of cure. Advanced or extensive atrophy or intestinal metaplasia, autoimmune gastritis, and defined hereditary syndromes require the appropriate surveillance or specialist assessment. Family history, migration background, and behavioral exposures inform further assessment but are not equivalent indications for surveillance. Population screening and targeted assessment should follow local disease burden, antimicrobial resistance, and endoscopy capacity.

## 1. Introduction

Although age-standardized rates of gastric cancer have declined over recent decades, the disease remains a leading cause of cancer death worldwide [1,2]. The highest disease burden is observed in East Asia, but inequality among countries extends beyond incidence alone. The mortality-to-incidence ratio is reported to be higher in countries with lower human development indices [1,2]. Population growth and aging can also increase the absolute number of cases even while individual risk decreases [2]. Therefore, disease control assessment should not be limited to incidence rates but must also encompass case counts, deaths, and the unequal distribution of these outcomes.

Gastric cancer is not a homogeneous disease. Cardia and non-cardia tumors differ in geographic distribution and exposure patterns [3]. Intestinal and diffuse types do not follow the same histological pathway or age pattern [4,5]. Molecular classification has clarified this heterogeneity, revealing subgroups with different behaviors, recurrence risks, and survival outcomes [6]. Consequently, the decline in intestinal-type or non-cardia cancer cannot be generalized to all forms of the disease. The increase or slower decline of early-onset cancer in some populations, especially young women, highlights the need to analyze trends by age, sex, subsite, and histology [7,8].

In a large portion of non-cardia cancers, chronic *Helicobacter pylori* infection initiates a chain of events progressing from inflammation to atrophy, intestinal metaplasia, dysplasia, and finally cancer [9,10]. However, only a subset of infected individuals develop cancer. Infection characteristics, host response, mucosal status, and behavioral and nutritional exposures collectively shape the disease pathway. Autoimmune gastritis, Epstein–Barr virus, tobacco, heavy alcohol consumption, high salt intake, and genetic susceptibility also play roles in different subgroups [11,12,13]. Thus, diagnosing infection alone is insufficient for individual risk estimation and must be interpreted alongside mucosal damage stage and host background.

The treatability of an infectious agent provides a crucial opportunity for primary prevention of gastric cancer. *Helicobacter pylori* eradication reduces cancer risk [14], but prevention is most effective when treatment occurs before severe atrophic gastritis develops. Even after the spread of atrophy or metaplasia, monitoring should continue [15,16]. In Japan and South Korea, endoscopy is part of population-level gastric cancer control programs [17,18], but implementing similar models in countries with different incidence rates and executive capacities requires separate evaluation [19].

Given this heterogeneity, prevention requires a pathway appropriate to the population and the individual. Age, sex, country of origin, migration history, family history, tobacco and alcohol exposure, *Helicobacter pylori* status, and the extent of atrophy or metaplasia inform assessment. Pathogenic variants may require a separate hereditary cancer pathway. The strength of evidence differs among these factors. Drug and nutritional associations are largely observational and may reflect reverse causality, treatment indications, or incomplete exposure measurement. Factors associated with risk should therefore be distinguished from established causes, markers used to guide assessment, and interventions shown to reduce cancer incidence.

Previous reviews have often examined different parts of this pathway separately. Some focused on global burden or incidence trends, others aggregated risk factors, and some evaluated only infection eradication, precancerous lesions, or screening. This fragmentation makes answering decision-making questions difficult, as it remains unclear which factor is most important at which stage of carcinogenesis, when intervention yields maximum benefit, and how much results from one strategy can be generalized to another population. Furthermore, global averages may obscure disease increases in certain subgroups and inequalities in access to prevention and early diagnosis.

This review examines gastric cancer incidence, mortality, temporal trends, survival, and differences by age, anatomy, histology, and molecular profile. It considers infectious, mucosal, behavioral, metabolic, and hereditary factors alongside evidence on *Helicobacter pylori* eradication, screening, and surveillance. The review integrates epidemiological patterns with prevention and screening evidence appropriate to disease stage. Findings are interpreted according to study design, population, and geographic applicability to inform strategies suited to baseline risk and healthcare capacity.

## 2. Materials and Methods

This narrative review was conducted through a comprehensive literature search of PubMed, Web of Science, Scopus, and Google Scholar from database inception to January 2026. No publication year restrictions were applied; only English-language publications were included. The search strategy was adapted to the structure of each database.

Searches were conducted in an iterative and topic-specific manner, consistent with the narrative nature of this review, which did not follow a pre-registered systematic protocol. Search terms combined “gastric cancer,” “stomach cancer,” or “gastric adenocarcinoma” with concepts related to epidemiology, incidence, mortality, survival, temporal trends, early-onset disease, anatomical subsite (cardia and non-cardia), risk factors, *Helicobacter pylori*, precancerous lesions (including atrophic gastritis and intestinal metaplasia), genetic susceptibility, eradication, screening, endoscopy, and prevention. Because the review addressed several interconnected aspects of gastric cancer, the combination of terms was adapted to each database and thematic focus rather than applying a single universal search strategy. The following represents the core conceptual search framework used across databases:

(“gastric cancer” OR “stomach cancer” OR “gastric adenocarcinoma”) AND (epidemiology OR incidence OR mortality OR survival OR trend OR “early onset” OR cardia OR noncardia OR “risk factor” OR “*Helicobacter pylori*” OR “precancerous lesion” OR “atrophic gastritis” OR “intestinal metaplasia” OR “genetic susceptibility” OR eradication OR screening OR endoscopy OR prevention).

Major bibliographic databases (including PubMed/MEDLINE, Scopus, and Web of Science) were searched, with search strategies tailored to the syntax and controlled vocabulary of each database. Google Scholar was used as a supplementary discovery source; the first several pages of results for key queries were screened, and reference lists of major reviews, authoritative guidelines, and index studies were hand-searched to identify additional relevant publications. No formal quantitative screening flow (e.g., number of records retrieved, screened, or excluded) was applied, as the selection process was narrative and guided by thematic relevance and the evolving scope of the review.

The iterative search identified publications that were considered during manuscript preparation. Sources were retained when they contributed directly to one or more thematic areas of the review and provided clinically or epidemiologically relevant information not adequately represented by other selected sources. Full-text articles were excluded primarily when they did not address the core thematic areas of the review (epidemiology, risk factors and progression, or prevention and screening), lacked sufficient clinical or epidemiological relevance, were duplicate reports of the same data, or provided only highly specialized technical details without broader contextual value. One reviewer conducted the literature search and selected the sources. Selection was not independently repeated, so there was no procedure for resolving disagreements between reviewers. Because this was an iterative narrative review rather than a protocol-driven systematic review, database-specific retrieval counts and a formal record-by-record exclusion log were not prospectively maintained.

Evidence was synthesized qualitatively with attention to study design, population, sample size when reported, outcomes, effect estimates, main findings, and limitations. Original studies were primarily used when discussing quantitative findings, whereas reviews, meta-analyses, and guidelines were used to assess consistency and provide broader clinical context.

The evidence was organized into three themes. The first covered epidemiological patterns, including incidence, mortality, temporal trends, survival, and differences by age, sex, anatomy, histology, and molecular profile. The second addressed factors related to risk and progression, including infection, mucosal damage, behavioral and nutritional exposures, metabolic status, social factors, genetic susceptibility, and drug exposures. The third covered *Helicobacter pylori* eradication, endoscopic or radiographic screening, and risk stratification. Due to heterogeneity in study designs, populations, outcomes, and effect estimates, no new meta-analysis was conducted.

Given the broad and heterogeneous scope of the evidence, this review was conducted as a narrative synthesis rather than a formal systematic review. Formal risk-of-bias appraisal using a standardized tool was not performed because the evidence comprised diverse study designs that could not be evaluated within a single framework; however, interpretation considered study design, sample size and representativeness, temporal precedence of exposure over outcome, exposure and outcome measurement, control of confounding, follow-up duration, estimate precision, and generalizability.

## 3. Results

### 3.1. Scope of the Narrative Synthesis

The review draws on the findings derived from 81 cited sources. Original studies and secondary analyses support the quantitative findings; previous reviews provide comparisons across studies; and guidelines and authoritative reports inform clinical and population recommendations. The sources were selected iteratively rather than as a prospectively fixed group of included studies. The bibliography records the material cited in the review and should not be interpreted as a screening total.

Primary outcomes included incidence, mortality, temporal changes, age and sex patterns, anatomical subsites, histological types, and survival. Infectious and behavioral factors, genetic susceptibility, precancerous lesions, effectiveness of *Helicobacter pylori* eradication, and endoscopic screening outcomes were also examined. These findings indicated that gastric cancer epidemiology cannot be explained by a single global trend, as changes in incidence and disease outcomes depend on age, sex, tumor subsite, genetic background, and the structure of early diagnosis programs.

### 3.2. Epidemiological Patterns of Gastric Cancer

#### 3.2.1. Incidence, Mortality, and Geographic Distribution

In 2022, an estimated 968,350 new gastric cancer cases and 659,853 deaths occurred worldwide, accounting for 4.9% of cancer cases and 6.8% of cancer deaths, respectively [1]. Gastric cancer ranked fifth for both incidence and mortality [1]. There were 627,229 cases in men and 341,121 in women. Age standardized incidence rates were 12.8 and 6.0 per 100,000, and mortality rates were 8.6 and 3.9 per 100,000 in men and women, respectively [1].

East Asia had the highest geographic concentration of the disease. This region accounted for 53.8% of new cases and 48.2% of global deaths in 2022 [8]. The mortality-to-incidence ratio was 0.9 in countries with low human development indices, compared to 0.7 in countries with very high indices [2]. Thus, geographic inequality was not limited to case numbers; the mortality burden relative to incidence also differed among countries.

The disease burden pattern differed by anatomical subsite. In the 2012 estimate, non-cardia cancer constituted 73% and cardia cancer 27% of cases. Non-cardia cancer was more common in 167 out of 184 countries. The male-to-female incidence ratio was approximately three-to-one for cardia cancer and two-to-one for non-cardia cancer [3]. In Iran, Finland, and Serbia, cardia cancer incidence was equal to or exceeded non-cardia cancer [3]. Consequently, male predominance and burden concentration in East Asia were stable patterns, but subsite shares varied within countries.

An Iranian registry analysis of 35,171 diagnoses during 2003 to 2008 also found male predominance, with average standardized incidence of 15.1 per 100,000 in men and 7.1 in women [20]. These historical estimates complement global comparisons without defining present incidence.

#### 3.2.2. Temporal Trends and Early-Onset Gastric Cancer

The cited analyses used different age categories. Tan et al. considered diagnosis before age 50 [8], Ning et al. used ages 15 to 49 [21], and Anderson et al. examined people younger than 50 [7]. Ning et al. also defined intermediate onset as ages 50 to 54 and later onset as age 55 or older [21]. These categories are interpreted within each study and are not pooled as identical groups.

Despite declines in age-standardized rates, the absolute number of cases increased. Between 1990 and 2017, approximately 356,000 new cases and 96,000 deaths were added to case counts. Over the same period, the age-standardized incidence rate decreased by 28%, and the age-standardized mortality rate decreased by 43.2% [22]. If 2020 rates remain constant, the annual number of new cases in 2040 will reach 1.77 million, and deaths will reach 1.27 million [2]. These two trends indicate that decreasing individual risk does not necessarily lead to a reduction in the absolute disease burden.

Incidence did not decrease at the same speed across all age groups. In 2022, there were 82,146 cases of gastric cancer in individuals under 50 years old, accounting for 8.48% of all cases. Deaths in this group were 51,963 [8]. Between 2003 and 2017, early-onset cancer incidence increased in men in three countries and women in eight countries. Increases occurred in both sexes in Australia, Colombia, and Ireland, while increases in Austria, Denmark, France, South Africa, and the United States were more pronounced in women [8].

In an analysis of 1990–2019, the annual decline in early-onset cancer incidence was 0.84%. This figure was 1.77% for the intermediate onset group, aged 50 to 54, and 1.10% for the later onset group, aged 55 or older [21]. Projections for 2020–2035 showed an annual increase of 1.44% in early-onset cancer incidence and relative stability in the intermediate onset trend [21]. In non-Hispanic whites in the US, non-cardia cancer incidence in individuals under 50 increased annually by 1.3%, while in those aged 50 and above, it decreased by 2.6% annually [7]. The increase was higher in young women, reaching 2.6% per year. The greatest growth was seen in stomach body tumors [7]. The overall decline therefore did not describe the experience of every age and sex group.

#### 3.2.3. Histological, Molecular Heterogeneity, and Survival

Among 54,099 registered cases, 74% of tumors were intestinal-type and 16% were diffuse-type [5]. From the 1978–1983 period to the 2001–2005 period, overall gastric cancer incidence decreased by 34%, and intestinal type incidence decreased by 44%. In contrast, diffuse-type incidence initially increased by 62% and then decreased slightly. Cardia cancer incidence remained nearly constant after a 23% increase [5]. Thus, the majority of the overall incidence drop was related to the decline in intestinal-type and non-cardia subsites.

Molecular classification revealed important clinical differences. The MSS/EMT subgroup occurred at a younger age, and over 80% of its tumors were diffuse-type. This subgroup was often diagnosed at stage III or IV, had a 63.4% recurrence rate, and exhibited the worst overall survival [6]. In contrast, the MSI subgroup was mostly associated with lower stages and the best prognosis. The association of MSS/EMT with poorer survival persisted after adjusting for clinical variables, with an HR of 1.899 [6].

Epstein–Barr virus was identified in 7.7% of gastric cancers. The likelihood of virus positivity was twice as high in diffuse tumors compared to intestinal tumors. Samples from the Americas had an OR of 2.3 compared to Asia [23]. In another study, EBV-associated cancers were more frequently accompanied by lymphoid stroma, higher lymphocyte infiltration, and greater expression of immune markers. Overexpression of HER2 was observed in only 6% of cases [24]. These findings indicated that EBV-associated gastric carcinoma is a distinct subgroup in terms of molecular and immune profiles, not a feature common to all tumors.

Survival improved over time, although this improvement was limited in advanced disease. In the US, five-year relative survival increased from 15% in 1975–1977 to 33% in 2012–2018 [25]. In the Netherlands, five-year survival for non-metastatic patients increased from 27.8% to 35.5% [26]. For metastatic patients, one-year survival increased from 9.9% to 19.3%, but three-year survival remained near 5% [26]. Short-term survival improvement in metastatic disease had not yet translated into sustained survival.

### 3.3. Factors Associated with Gastric Cancer Risk and Progression

#### 3.3.1. Infection, Mucosal Damage, and Precancerous Lesions

The strongest association among infectious factors was observed with *Helicobacter pylori*. In a large Chinese cohort, seropositivity was associated with an HR of 5.94 for non-cardia cancer and 3.06 for cardia cancer. The population-attributable fraction of infection for these two subsites was estimated at 78.5% and 62.1%, respectively [9]. Thus, the effect size was larger for non-cardia cancer, but the association was not limited to this subsite alone.

Mucosal status provided more information than infection diagnosis alone. In a twenty-year follow-up, incomplete intestinal metaplasia had an OR of 13.4 compared with complete type, with a wide 95% confidence interval of 1.8 to 103.8. High-risk OLGA and OLGIM stages were associated with cancer risks of 26.4 and 19.4, respectively [10]. In a case–control study, the OR for gastric xanthoma was 2.64 and for gastric ulcer was 5.58 [27]. Pernicious anemia was also associated with an OR of 2.75 for all gastric cancer and 3.91 for non-cardia cancer. After excluding this disease, the overall association of other autoimmune disorders with gastric cancer disappeared [11]. See Table 1.

#### 3.3.2. Behavioral, Nutritional, and Metabolic Factors

In young individuals, tobacco and alcohol were associated with an increased risk of early-onset cancer. Compared to never-smokers, the HR was 1.121 for former smokers and 1.282 for current smokers [29]. Hazardous alcohol consumption had an HR of 1.212. The highest risk was observed in individuals who were current smokers and hazardous alcohol consumers, with an HR reaching 1.447 [29]. In individuals with a history of *Helicobacter pylori* eradication, heavy cigarette smoking, daily consumption of at least 30 g of alcohol, and abdominal obesity were associated with HRs of 1.34, 1.23, and 1.11, respectively [30]. The persistence of these associations after eradication indicated that eliminating infection does not remove all risk.

Constant addition of salt to food was associated with an HR of 1.41. This association remained after excluding cases diagnosed in the first year and individuals with recorded infection history [31]. In a case–control study, consuming salty foods at least three times a week had an OR of 2.22. Consuming less than 100 g of fresh fruit per day was associated with an OR of 5.55, although this estimate was derived from a single hospital population [27].

The relationship between Body Mass Index (BMI) and risk depended on the tumor subsite. An increase of five units in BMI was associated with an HR of 1.15 for cardia cancer, but no specific association was seen for non-cardia cancer. Overweight or obesity also had an HR of 1.24 for cardia cancer [32]. In a national cohort, a BMI less than 18.5 compared to the range of 23–24.9 was associated with an OR of 1.44. Fasting glucose of at least 126 mg/dL or diabetes showed a weaker association with an OR of 1.09, which was more prominent in women [33]. These results did not support a linear and uniform relationship between general obesity and all types of gastric cancer.

Social inequality was also evident at the individual level. In non-Western immigrant women residing in Northern European countries, the RR of gastric cancer incidence was 1.68 and the RR of gastric cancer mortality was 1.49 compared to non-immigrant women. Conversely, university education compared to primary education was associated with an incidence RR of 0.69 and a mortality RR of 0.62 [34]. The persistence of these differences after migration indicated that reducing national rates does not necessarily lead to equal risk distribution. These associations identify a social gradient and do not establish education as an independently tested preventive intervention.

#### 3.3.3. Genetic Susceptibility and Heredity

In families with pathogenic *CDH1* mutations, the cumulative risk of gastric cancer by age 80 was estimated at 70% for men and 56% for women. Variants in *CTNNA1*, *ATM*, *PALB2*, *BRCA2*, and several other susceptibility genes were also identified in families without this mutation [13]. Lynch syndrome carriers had an age-standardized incidence ratio of 3.4. The lifetime risk of gastric cancer was 8% in male carriers and 5.3% in female carriers, with the highest gene-specific risk observed in *MSH2* carriers [35].

Among 3706 patients with gastric cancer referred for multigene testing, 13.3% carried at least one pathogenic or likely pathogenic germline variant [48]. A separate international study of 750 patients selected for early onset disease or a family history of cancer identified such variants in 6%. Only 16 of the 45 carriers met the assessed genetic testing criteria [49]. These proportions describe selected clinical populations and should not be interpreted as the prevalence among all patients with gastric cancer.

In early-onset cancer, loss-of-function variants in *CDH1* and *ATM* were enriched. At the replication stage, the OR of gastric cancer in carriers of the pathogenic *ATM* variant was 3.94 [36]. Truncating *CTNNA1* variants also had an OR of 7.0, although their penetrance was lower than pathogenic *CDH1* variants [37]. In total, genetic susceptibility was not limited to diffuse cancer and the *CDH1* gene, and some carriers also lacked an obvious family pattern.

#### 3.3.4. Drug Exposures

In a national cohort, the HR of gastric cancer for aspirin was 0.72 and for statins was 0.67. The HR of 0.85 for metformin was not statistically significant [39]. In diabetic patients followed up after *Helicobacter pylori* eradication, the HR for metformin use was 0.49, decreasing to 0.35 with use of at least three years [40]. The difference in these results suggested that the metformin association might depend on population characteristics, duration of use, or infection status.

Long-term use of proton pump inhibitors, after establishing an appropriate time lag between exposure and diagnosis, was not associated with an increase in non-cardia cancer, and an adjusted OR of 1.01 (95% CI 0.96 to 1.07) was reported. The positive association was only seen in models that included short-term use, the period close to diagnosis, or variables related to indication bias [41]. In another analysis, excluding the last year of use before diagnosis did not confirm increased risk [42]. This pattern was more consistent with reverse causality and indication bias than with an independent drug effect.

### 3.4. Prevention and Early Diagnosis Patterns

#### 3.4.1. *Helicobacter pylori* Eradication

Detection of current infection and confirmation of eradication serve different purposes. Serology can indicate exposure, but antibodies may persist after treatment and cannot establish cure. Eradication should be confirmed with a test of active infection, usually a urea breath test or a validated stool antigen test. Testing should be performed after a suitable interval following treatment, generally 4 to 6 weeks without antibiotics or bismuth and after withholding proton pump inhibitors for 14 days. Histology can contribute when endoscopy is otherwise indicated. Previous exposure, persistent infection, and confirmed eradication should not be treated as interchangeable when interpreting cancer risk [15].

The choice of eradication regimen should reflect local resistance patterns, previous antibiotic use, and susceptibility results when available. Confirmation of cure distinguishes successful treatment from persistent infection and informs further management. Population programmes also require monitoring of resistance, adherence, and treatment outcomes. Repeated empirical use of ineffective regimens should be avoided [15].

In one population program, *Helicobacter pylori* prevalence decreased from 64.2% to 15%, and reinfection rate remained less than one percent per person-year. This program was associated with a 53% reduction in gastric cancer incidence, while a 25% reduction in mortality was not statistically significant [43]. During the same period, atrophic gastritis prevalence decreased from 55.9% to 15.9%, and intestinal metaplasia prevalence decreased from 31.7% to 21.4% [43].

In a randomized placebo controlled trial after endoscopic resection of early gastric cancer or high grade adenoma, metachronous cancer occurred in 7.2% of treated patients and 13.4% of patients receiving placebo over a median 5.9 years. The HR was 0.50 (95% CI 0.26 to 0.94) in the modified intention to treat analysis [45]. In a separate trial involving infected first degree relatives of patients with gastric cancer, cancer developed in 1.2% of the treatment group and 2.7% of the placebo group over a median 9.2 years. The HR was 0.45 (95% CI 0.21 to 0.94) [44]. These trials addressed different clinical populations and outcomes, so their estimates should be interpreted within those settings.

The effect of eradication became more apparent over time. In individuals with evidence of infection, eradication performed at least six years prior to assessment had an HR of 0.44 compared to no eradication. A temporary increase in cancer diagnosis was observed in the first year [50]. In a twenty-year follow-up, sustained clearance of infection prevented the progression of atrophic gastritis. However, reversal of intestinal metaplasia, except in mild stages, was limited [10].

#### 3.4.2. Endoscopic and Radiographic Screening

Screening was associated with a higher likelihood of diagnosing cancer at a localized stage. Among individuals with screening history, 53.8% of tumors were localized. This ratio was 40.6% in unscreened individuals [18]. Compared to no screening, the OR of localized tumor diagnosis was 2.10 for endoscopy and 1.24 for upper gastrointestinal imaging. The probability of localized diagnosis also increased with more frequent screening [18].

In a national analysis, any screening was associated with an OR of 0.79 for gastric cancer death. The OR of death was 0.53 for endoscopy, 0.98 for upper gastrointestinal imaging, and 0.45 for receiving both methods [46]. In individuals who underwent endoscopy three or more times, the OR of death decreased to 0.19 [46]. In another population cohort, the HR of cancer death was 0.39 for endoscopy and 0.63 for radiography. The HR of advanced disease incidence was reported as 0.78 and 0.88 for these two methods, respectively [47]. In direct comparison, endoscopy had an RR of 0.327 for gastric cancer death compared to radiographic screening [51]. See Table 2.

#### 3.4.3. Risk Stratification for Targeted Screening

A Chinese multicentre study of 14,929 adults attending screening gastroscopy evaluated a score based on age, sex, pepsinogen I/II ratio, gastrin 17, *Helicobacter pylori* status, and pickled and fried food consumption. Participants were randomly divided into derivation and validation groups. Cancer prevalence in the derivation group was 1.2%, 4.4%, and 12.3% in the low-, intermediate-, and high-score groups, respectively [52]. Restricting endoscopy to the two higher groups would have detected 70.8% of all cancers and 70.3% of early cancers while reducing the number of endoscopies by an estimated 66.7%. Negative predictive values were 98.8% and 98.7% in the derivation and validation groups, respectively [52]. This was an estimate within a screening population, not a demonstrated reduction in endoscopy use or cancer mortality in clinical practice.

Combining infection markers and mucosal damage markers improved risk discrimination. In individuals without a history of eradication, simultaneous or separate positivity of *Helicobacter pylori* test and pepsinogen was associated with an HR of 5.89. The highest risk was observed in individuals negative for infection and positive for pepsinogen, with an HR of 8.79 [50]. This pattern was consistent with antibody reduction following progression of mucosal damage. Therefore, a negative infection test alone was not sufficient to place an individual in the low-risk group [50].

**Figure 1:**
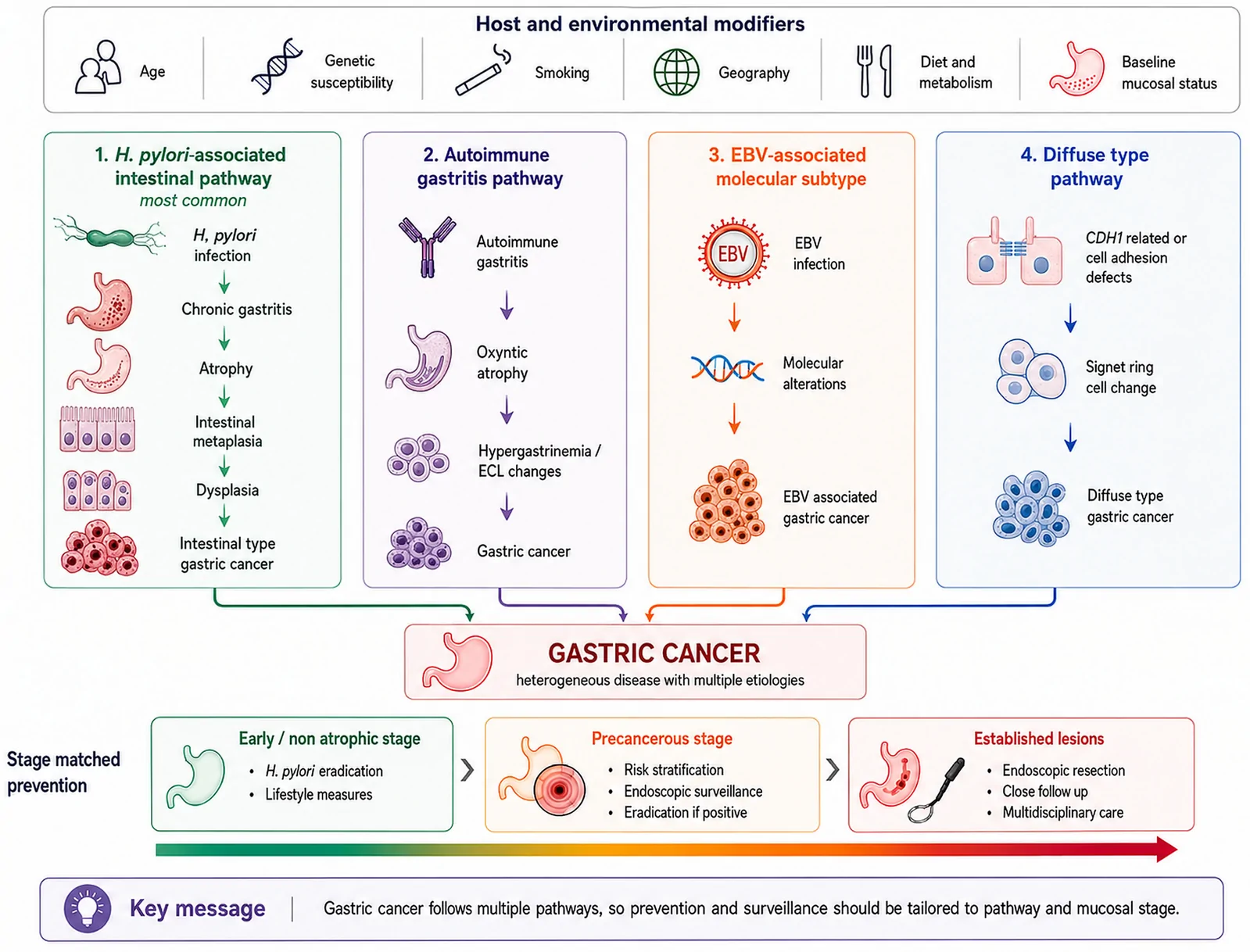
Heterogeneous pathways of gastric carcinogenesis and stage-matched prevention. The dominant *Helicobacter pylori*-associated mucosal sequence is shown alongside autoimmune gastritis, EBV-associated gastric carcinoma, and diffuse-type pathways. Host, environmental, and genetic modifiers influence progression, while prevention and surveillance are matched to mucosal stage and residual risk. In autoimmune gastritis, ECL changes indicate a separate neuroendocrine pathway. EBV, Epstein Barr virus; ECL, enterochromaffin like cells.

The prevention evidence supports treatment of infection alongside assessment of mucosal damage, hereditary susceptibility, and other exposures. Eradication offers preventive benefit, but it does not remove established mucosal risk or replace indicated surveillance. Endoscopic screening is associated with earlier diagnosis and lower mortality, although its effect estimates remain dependent on study design and setting. Figure 1 presents the *Helicobacter pylori* associated intestinal sequence alongside autoimmune gastritis, EBV associated gastric carcinoma, and diffuse type disease. These pathways do not represent a single sequence followed by all gastric cancers.

## 4. Discussion

Declining global averages conceal clinically distinct patterns by age, subsite, histology, molecular profile, and health-system capacity. Reviews of this heterogeneity argue against applying a single preventive pathway to all populations [53,54]. Falling age-standardized incidence can coexist with rising case counts as populations grow and age, so current rates alone should not define future service needs [2,55,56]. Reports of increasing early-onset gastric cancer, especially among young women in some countries, deserve attention but do not establish a uniform global epidemic. Small case numbers, incomplete subsite registration, and changes in coding may explain part of the pattern, although findings across several analyses argue against dismissing it as registration error alone [7,8,21]. Proposed explanations such as microbiome change and greater recognition of autoimmune gastritis remain hypotheses because registry studies do not measure them directly [7]. Longitudinal cohorts with individual data on infection, autoimmunity, and precise tumor subsite are needed.

Anatomical and histological heterogeneity also explains some disagreement among risk-factor studies. Obesity is more consistently associated with cardia cancer than with non-cardia or all gastric cancer [32,57], while the weaker diabetes signal is also largely subsite-specific [58]. Analyses that combine all tumors may therefore dilute a true subgroup association or misattribute it to the entire disease. Future studies should report subsite, Lauren type, and molecular features together [3,5]. Survival trends require similar caution. Randomized trials support benefit from adjuvant therapy after resection [59,60], but improved population survival can also reflect stage migration and earlier detection. Stage-specific survival and cause-specific mortality are therefore more informative than crude overall survival when comparing periods or countries [26,53].

An Iranian systematic review describes differences between provinces and variation in the measures and designs used to study epidemiology and risk factors [61]. These findings support assessment below national averages. They provide regional context and should not be treated as current provincial estimates or as an independent test of screening effectiveness.

Among causal factors, evidence regarding *Helicobacter pylori* is stronger than other factors in terms of epidemiological consistency, temporality, biological plausibility, and response to intervention. However, the high prevalence of infection and the small proportion of infected individuals developing cancer indicate that the presence of bacteria alone is insufficient for individual prediction. Infection duration, strain characteristics, host immune response, location and severity of inflammation, diet, and mucosal damage stage all affect the final outcome. Laboratory studies have shown that virulence factors such as VacA can enhance immune tolerance and infection persistence, and salt consumption may increase colonization, atrophy, and epithelial proliferation. These studies do not directly measure human cancer but provide a biological explanation for the population associations between infection and salt [62,63]. Thus, infection creates an inflammatory background, but progression to cancer depends on host-environment interaction.

A negative infection test does not always mean the individual has never been exposed. With the progression of atrophy and metaplasia, bacterial density and serologic response may decrease, potentially placing an individual with severe mucosal damage in the negative group. This misclassification biases the association between infection and cancer toward the null value and can explain the seemingly higher risk in individuals who are infection-negative but pepsinogen-positive. Reclassifying likely false-negative cases has created estimates closer to prospective studies [50,64]. Therefore, risk assessment should not be limited to a single cross-sectional test and must include eradication history, evidence of atrophy, pepsinogen ratio, and if endoscopy is performed, the extent and type of metaplasia.

Bacterial infection is not the only factor creating a high-risk mucosal background. The association of pernicious anemia with non-cardia cancer and metachronous neoplasia suggests a separate autoimmune pathway [11,28]. However, generalizing this risk to all autoimmune diseases is inaccurate. In a population-based study, the overall association of other autoimmune diseases with gastric cancer disappeared after excluding pernicious anemia [11], whereas a meta-analysis reported a weaker but still significant association after the same exclusion [65]. Differences in disease definitions, populations, and adjustment for infection status may contribute to this discrepancy, but the cited studies do not resolve it. Epstein–Barr virus also shows another pathway, associated with lymphoid microenvironment, expression of immune markers, and specific molecular patterns [23,24]. Thus, viral infection represents a distinct biological category rather than a common risk factor for all gastric cancers.

Evidence regarding behavioral factors is not equally strong. Tobacco use has shown a relatively stable and dose-dependent association in various populations and meta-analyses, making it one of the most reliable modifiable factors [66,67]. Regarding alcohol, a distinction must be made between any consumption and heavy consumption, as the association is more apparent at high levels and when combined with tobacco [12,29]. Regarding salt, the concordance of prospective studies, pooled analyses, and laboratory evidence strengthens the biological argument [12,62,68], although geographic heterogeneity and indirect measurement of consumption make precise risk estimation difficult. These factors likely have additive and interactive effects. Therefore, controlling tobacco, reducing heavy alcohol consumption, and limiting salt must be done alongside infection control, not instead of it.

Nutritional and metabolic associations require more cautious interpretation. Dietary assessment usually relies on individual recall, different definitions of consumption amounts, and correlated lifestyle patterns. In case–control studies, individuals may change their diet in response to preclinical symptoms, reversing the direction of association. Umbrella reviews have shown that despite many significant results, only a small fraction of factors reach high epidemiological credibility, and heterogeneity, publication bias, and poor quality of initial reviews are common in this field [69,70]. Regional differences in salt and some food associations may result from food preservation methods, simultaneity with infection, and overall dietary pattern [12]. Therefore, recommending a healthy dietary pattern is justified, but evidence does not support introducing a specific food item or supplement as a definitive preventive agent.

Drug-related findings show how much study design affects results. Meta-analyses of retrospective studies between proton pump inhibitors and gastric cancer reported a positive association, but high heterogeneity, inadequate control of infection, and immortal time bias limited its interpretation [71]. In analyses that allowed for sufficient time lag between exposure and diagnosis and more precisely controlled for indication bias and underlying diseases, this association disappeared. Such a change is consistent with reverse causality, as undiagnosed tumor symptoms or precancerous disease may themselves initiate drug use [41,42]. The inverse associations reported for aspirin, statins, and metformin are also insufficient for recommending drug prevention, as the healthy user effect, comorbidity differences, concurrent medication use, and lack of information on mucosal damage could explain these results [39,40,72]. Decisions to start or stop these drugs should be based on primary indications and overall benefit–risk balance, not on observational associations with gastric cancer.

Genetic findings have moved beyond the classic pattern of hereditary diffuse cancer, showing a wider spectrum of susceptibility genes. The role of *CDH1* is established, but penetrance estimates in multiple-case families may overestimate risk for carriers identified in the general population [13]. Newer data identify *CTNNA1* as a gene with moderate penetrance and support the role of *ATM* and some DNA-repair genes in early-onset or familial cancer [36,37]. However, the high yield of multi-gene tests in patients referred for testing cannot be generalized to all patients, as referral bias, panel size differences, and lack of histological information are common in these studies [48,49]. These findings support further evaluation of existing testing criteria, but decisions about universal testing require prospective data from consecutive patients, specific penetrance estimates for each gene, and proof of clinical benefit of care based on test results.

Molecular classification links age, histological, recurrence, and survival differences to tumor biology. In existing data, Epstein–Barr virus-associated cancers were accompanied by lymphocytic infiltration and greater expression of immune markers, while the MSI subgroup showed the best prognosis [6,24]. In contrast, the MSS/EMT phenotype was more associated with diffuse type, peritoneal recurrence, and poor prognosis [6]. Different molecular systems do not fully overlap [73], and the cost of testing and temporal/spatial heterogeneity of markers can limit their application [6,74]. Currently, MSI, HER2, PD-L1, and CLDN18.2 are more important for prognosis and treatment selection than for population screening. The next important question is whether adding molecular markers to infection information, mucosal status, and family history can better predict risk before cancer develops, rather than just classifying existing tumors.

The most important preventive message of this review was the benefit of *Helicobacter pylori* eradication. The direction of effect was consistent across trials, cohorts, population programs, and new meta-analyses [14,75], but the magnitude of benefit depended on the timing and stage of intervention. Guidelines consider the greatest preventive capacity before severe atrophy develops, and long-term follow-up has shown that after infection clearance, atrophy is more reversible than intestinal metaplasia [10,15]. In patients treated for primary neoplasia, eradication reduced metachronous cancer [45], but it did not replace the need for endoscopic monitoring of damaged mucosa [16]. Therefore, the point of no return should not be considered an absolute boundary. Late intervention is still beneficial, but alone it does not return risk to baseline levels.

This evidence applies in population programs when baseline risk and execution quality are considered. A uniform relative reduction in a high-risk region can prevent a significant number of cancers, whereas in a low-risk population, many more individuals would need to be tested and treated. Real-world effectiveness depends on participation, test accuracy, selection of antibiotic regimens appropriate for antimicrobial resistance, confirmation of successful eradication, and prevention of reinfection. Since most trials were conducted in East Asia, the exact magnitude of benefit is not yet generalizable to other regions [14]. Therefore, the test-and-treat strategy must be accompanied by local epidemiology and antimicrobial resistance monitoring. International consensus guidelines also recommend eradication but emphasize continued monitoring of individuals with advanced atrophy or metaplasia [15].

The association of endoscopic screening with reduced advanced disease and mortality has been replicated in various studies, strengthening the likelihood of a real effect. However, observational studies may overestimate this effect, as screened individuals typically have better health awareness, access, and treatment adherence, and statistical adjustment may not fully eliminate these differences. Earlier diagnosis can also increase calculated survival without changing the time of death, and identifying slow-growing tumors creates length-time bias. Therefore, cause-specific mortality and incidence of advanced disease are more valid measures than the ratio of early-stage cancers or post-diagnosis survival [47]. Japanese guidelines, despite recommending endoscopy, have primarily regarded the evidence as observational and reported uncertainty regarding screening intervals, stopping age, and overdiagnosis [17].

The most important question for policy-making is the generalizability of the East Asian experience to other regions. In moderate-risk areas, evidence supports the ability of endoscopy to detect early lesions, but mortality data come from a limited number of studies and a single geographical context, and endoscopic techniques, sampling methods, and definitions of precancerous lesions have not been uniform [19]. Therefore, this evidence is insufficient to justify universal screening in a low-risk country. Cost-effectiveness models show that targeted endoscopy combined with selective monitoring can be cost-effective in high-risk groups, but the result depends on metaplasia prevalence, service costs, endoscopy quality, and progression probability [76,77]. Transferring a national program without assessing local risk and capacity may divert resources from those who benefit most.

Implementation also depends on whether eligible people attend. A systematic review of 13 studies identified absence of symptoms, fear of the procedure or its result, cost, and embarrassment among reported barriers to gastric cancer screening [78]. Programmes should assess these obstacles locally when planning invitations and access. The review explains participation barriers and does not establish a mortality benefit from any specific intervention.

Risk assessment may help select individuals for endoscopy, but the available prediction scores should not be treated as complete prevention strategies. The Chinese score identified a higher prevalence of cancer in selected groups, while the estimated reduction in endoscopy would have left some cancers undetected [52]. Its performance requires evaluation outside the population in which it was developed. Mucosal stage remains important when deciding on surveillance, including advanced OLGA or OLGIM stages [16]. Assessment should therefore begin with infection history and background risk, consider evidence of atrophy or metaplasia, and lead to endoscopy when the residual risk warrants it. A negative *Helicobacter pylori* test alone does not establish low risk when mucosal damage is present. After eradication, follow up should continue to reflect mucosal findings and the relevant clinical guidance.

Migration patterns show that risk is not solely dependent on current residence. Higher incidence in first-generation migrants and the persistence of part of this increase in the next generation are consistent with early-life exposures and the long duration of carcinogenesis [79]. In non-Western immigrant women in Northern European countries, higher education was associated with lower incidence and mortality, but the data did not clarify the mechanism of this social gradient [34]. Therefore, a policy based only on national averages may ignore high-risk populations residing in a low-risk country. Country of origin, migration generation, and individual risk characteristics must be included in targeted programs. At the same time, broad racial categories should not replace etiology, as heterogeneity within these groups is significant [77,79]. The goal should be identifying actual exposure and risk, not solidifying demographic labels.

At the population level, the approach should reflect local disease burden and service capacity. In high incidence regions, organized infection control and quality endoscopy may be appropriate where participation, antimicrobial resistance, and treatment outcomes can be monitored. In intermediate incidence regions, assessment can use age, family history, country of origin, infection status, and mucosal findings to select individuals for endoscopy. In low incidence regions, assessment should focus on hereditary families, advanced precancerous lesions, autoimmune gastritis, and people who migrated from regions with a high burden. Suspected hereditary cancer requires the appropriate genetic evaluation [80]. The 2025 MAPS III guideline distinguishes population screening from surveillance of established mucosal disease. It supports screening in high-incidence regions, conditional consideration in intermediate-incidence regions where cost-effectiveness is demonstrated, and no population screening in low-incidence regions. It also recommends surveillance for advanced atrophy or metaplasia and suggests endoscopic follow up for autoimmune gastritis, with decisions reflecting age, comorbidity, and mucosal findings [81]. These recommendations do not make family history, migration, or behavioral exposures equivalent independent indications for surveillance.

The interpretation of evidence differs by the question being addressed. Registry and burden studies describe disease patterns. Observational studies identify associations but may not establish causes or intervention effects. Randomized eradication trials provide direct evidence of prevention in their studied populations. These distinctions determine how the results inform clinical and population recommendations.

The narrative design limits reproducibility and leaves the review open to selection bias. Source selection was performed by one reviewer, search and exclusion counts were not prospectively recorded, and no formal risk of bias assessment or certainty grading was undertaken. The qualitative interpretation cannot replace those procedures. Studies also differed in age definitions, tumor subsites, exposure measurement, outcomes, and control of confounding. Much of the screening and eradication evidence comes from East Asia, and registry coverage varies among regions. These limitations restrict the transfer of numerical estimates and prevent a universal screening rule from being inferred from this review.

Further studies should use consistent age definitions and report infection history, tumor subsite, histology, and sex when examining younger populations. Screening programmes and risk scores require evaluation outside East Asia using gastric cancer mortality and advanced disease outcomes. Eradication studies should account for local antibiotic resistance and confirmed treatment success. Drug studies need a sufficient interval between exposure and diagnosis and careful consideration of treatment indications. Genetic studies should distinguish estimates from families or referred patients from those obtained in unselected populations. More complete cancer registration remains necessary in regions with limited coverage.

## 5. Conclusions

Declining standardized rates do not remove the need for gastric cancer prevention. Population growth and aging can increase case numbers despite falling rates, and survival in advanced disease remains limited. Reported increases in some younger populations require attention but should not be interpreted as a uniform global epidemic. Monitoring should distinguish age, sex, anatomical subsite, and histology, while programme evaluation should include advanced disease and mortality.

Prevention should include identification and treatment of *Helicobacter pylori* with confirmation of eradication. Advanced or extensive atrophy or intestinal metaplasia warrants surveillance according to the relevant guidance. Autoimmune gastritis and defined hereditary syndromes require their own assessment and follow up pathways. Family history, migration background, tobacco use, and heavy alcohol exposure inform additional assessment but are not interchangeable with established surveillance indications. Population screening is most defensible in settings with a high disease burden and the capacity to provide effective treatment and quality endoscopy. In intermediate and low incidence settings, selection should reflect individual risk, mucosal findings, local costs, and service capacity.

Observational drug associations do not justify starting aspirin, statins, or metformin solely to prevent gastric cancer. Proton pump inhibitor findings require consideration of reverse causality and treatment indications. A practical approach combines infection control and reduction in harmful exposures with mucosal assessment, hereditary evaluation when indicated, and appropriate endoscopic follow up. Its use should follow existing clinical guidance, and any proposed prediction score or screening programme requires evaluation in the population where it will be applied. Effect estimates derived mainly from East Asia should not be transferred unchanged to other healthcare systems.

## Figures and Tables

**Table 1:** Evidence Types and Interpretation of Gastric Cancer Factors and Interventions.

Domain	Factor, Marker, or Intervention	Evidence Type	Interpretation
Infectious and mucosal	*Helicobacter pylori* infection, particularly for non-cardia cancer [9]	Established causal factor and intervention target	Higher risk, strongest for non-cardia disease; infection alone does not determine progression.
	Pernicious anemia and autoimmune gastritis [11,28]	Distinct mucosal risk pathway	Higher risk; the finding should not be generalized to all autoimmune disorders.
	Incomplete intestinal metaplasia and advanced OLGA or OLGIM stages [10]	Risk marker and surveillance indication	Higher residual risk that reflects established mucosal damage.
Tumor subtype	EBV-associated gastric carcinoma [23,24]	Molecular and etiologic subtype	A distinct subtype rather than a conventional exposure.
Lifestyle and metabolic	Tobacco use and heavy alcohol consumption [29,30]	Established tobacco risk; observational alcohol association	Higher risk, particularly with heavy or combined exposure.
	High salt intake and low fresh fruit intake [27,31]	Observational dietary associations	Higher risk; exposure measurement and regional context vary.
	Body mass index and fasting glucose [32,33]	Subsite or subgroup associations	Cardia-specific or selected-group signals; residual confounding remains possible.
Social	University education compared to primary education [34]	Social risk marker	Inverse association; education is not a preventive intervention.
Hereditary	Pathogenic variants in *CDH1*, *CTNNA1*, *ATM*, *MLH1*, and *MSH2* [13,35,36,37]	Hereditary susceptibility	Higher risk; penetrance and testing yield vary by gene and setting.
Pharmacological	Aspirin, statins, metformin, and proton pump inhibitors [38,39,40,41,42]	Observational drug associations	Mixed or inverse findings; lagged PPI analyses show no independent increase. These results do not justify changing treatment.
Prevention	Successful eradication of *Helicobacter pylori* [43,44,45]	Preventive intervention	Lower incidence, with greatest benefit before advanced mucosal damage; residual risk persists.
Early detection	Endoscopic screening [18,46,47]	Observational early detection evidence	Earlier stage and lower mortality associations; screening bias remains possible.
	Upper radiographic screening [46,47]	Observational early detection evidence	Mixed or weaker associations that vary by program and population.

Note: Evidence types distinguish established causal factors and interventions from risk markers and observational associations.

**Table 2:** Selected evidence for prevention and early detection. The table reports study design, population, comparator, and outcome. The eradication trials were randomized, the population programme used historical controls, and the screening and prediction studies were observational.

Intervention	Study Design	Population and Comparator	Main Outcome	Reported Estimate
*Helicobacter pylori* eradication	Population cohort with historical controls	Matsu Islands, Taiwan; 6512 participants aged 30 or older; 85.5% coverage; programme compared with the 1995 to 2003 historical period	Reduced incidence of gastric cancer	Incidence reduction: 53% (95% CI 30 to 69%); mortality reduction: 25% (95% CI minus 14 to 51%; *p* = 0.18) [43]
	Double-blind randomized placebo-controlled trial	Korea after endoscopic resection; 470 randomized; 396 analyzed; eradication versus placebo; median follow up 5.9 years	Reduction in metachronous cancer	HR 0.50 (95% CI 0.26 to 0.94) [45]
	Double-blind randomized placebo-controlled trial	Korea; infected first degree relatives; 1838 randomized; 1676 analyzed; eradication versus placebo; median follow up 9.2 years	Reduced incidence of gastric cancer	HR 0.45 (95% CI 0.21 to 0.94) [44]
Endoscopic screening	Retrospective matched analysis	Korean national programme; 8044 screened and 8044 unscreened patients	Increased diagnosis at localized stage	OR 2.10 (95% CI 1.90 to 2.33) [18]
	Nested case–control study	Korean national programme; 54,418 fatal gastric cancer cases and 217,672 matched controls; endoscopy versus no screening	Reduced gastric cancer mortality	OR 0.53 (95% CI 0.51 to 0.56) [46]
Endoscopy compared with radiographic screening	Observational population cohort	Japan; 9950 endoscopy and 4324 radiography attendees aged 40 to 79; mortality follow up through 2013	Reduced gastric cancer mortality	RR 0.327 (95% CI 0.118 to 0.908) [51]
Risk score before endoscopy	Multicentre cross-sectional study with derivation and split-sample validation	China; 14,929 adults aged 40 to 80 attending screening gastroscopy; derivation and validation groups in a 2:1 ratio	Sensitivity 70.8%; estimated endoscopy reduction 66.7%; cancer present at screening	AUC 0.76 in derivation and 0.73 in validation [52]

Note: The randomized eradication findings and observational screening associations provide different levels of support for intervention. Screening estimates may be affected by differences between attendees and nonattendees, lead time, and length time bias. The risk score was assessed against cancer present at screening and does not establish a mortality benefit or universal thresholds for endoscopy.

## Data Availability

No new data were created or analyzed in this study. Data sharing is not applicable to this article.
